# Miniaturized planar Si-nanowire micro-thermoelectric generator using exuded thermal field for power generation

**DOI:** 10.1080/14686996.2018.1460177

**Published:** 2018-05-24

**Authors:** Tianzhuo Zhan, Ryo Yamato, Shuichiro Hashimoto, Motohiro Tomita, Shunsuke Oba, Yuya Himeda, Kohei Mesaki, Hiroki Takezawa, Ryo Yokogawa, Yibin Xu, Takashi Matsukawa, Atsushi Ogura, Yoshinari Kamakura, Takanobu Watanabe

**Affiliations:** a Faculty of Science and Engineering, Waseda University, TokyoJapan; b Graduate School of Science and Technology, Meiji University, Kawasaki, Japan; c National Institute for Materials Science, Tsukuba, Japan; d National Institute of Advanced Industrial Science and Technology, Tsukuba, Japan; e Graduate School of Engineering, Osaka University, Osaka, Japan; f JSPS Research Fellow DC, Tokyo, Japan

**Keywords:** Energy harvesting, thermoelectric generator, Si-nanowire, exuded thermal field, parasitic thermal resistance, 50 Energy Materials, 105 Low-Dimension (1D/2D) materials, 206 Energy conversion / transport / storage / recovery, 210 Thermoelectronics / Thermal transport / insulators, 306 Thin film / Coatings, 503 TEM, STEM, SEM

## Abstract

For harvesting energy from waste heat, the power generation densities and fabrication costs of thermoelectric generators (TEGs) are considered more important than their conversion efficiency because waste heat energy is essentially obtained free of charge. In this study, we propose a miniaturized planar Si-nanowire micro-thermoelectric generator (SiNW-μTEG) architecture, which could be simply fabricated using the complementary metal–oxide–semiconductor–compatible process. Compared with the conventional nanowire μTEGs, this SiNW-μTEG features the use of an exuded thermal field for power generation. Thus, there is no need to etch away the substrate to form suspended SiNWs, which leads to a low fabrication cost and well-protected SiNWs. We experimentally demonstrate that the power generation density of the SiNW-μTEGs was enhanced by four orders of magnitude when the SiNWs were shortened from 280 to 8 μm. Furthermore, we reduced the parasitic thermal resistance, which becomes significant in the shortened SiNW-μTEGs, by optimizing the fabrication process of AlN films as a thermally conductive layer. As a result, the power generation density of the SiNW-μTEGs was enhanced by an order of magnitude for reactive sputtering as compared to non-reactive sputtering process. A power density of 27.9 nW/cm^2^ has been achieved. By measuring the thermal conductivities of the two AlN films, we found that the reduction in the parasitic thermal resistance was caused by an increase in the thermal conductivity of the AlN film and a decrease in the thermal boundary resistance.

## Graphical abstract

**Figure:**
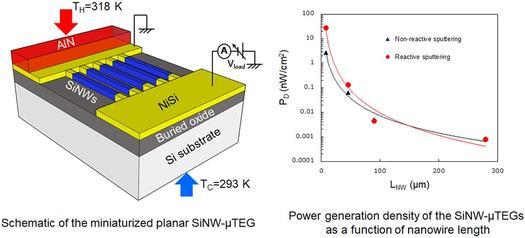

## 1. Introduction

Wireless power supply is a critical issue in realizing the Internet of Things (IoT) society, in which sensing, data collection, and communication would be performed using a trillion-sensor network [1]. To date, most wireless power supplies have used batteries in various forms [2]. However, batteries suffer from various limitations. For example, batteries often dominate the size, and sometimes the cost, of devices. In addition, batteries must be replaced or recharged at the end of their lifetime, which should be avoided in many cases, such as those involving the structural health monitoring of bridges, tunnels, and buildings; and implanted medical devices, such as those used for monitoring the blood-glucose levels of diabetic patients [2,3]. The use of energy harvesting devices that extract energy from the environment is a promising approach to solve these problems. Primarily, there are four forms of energy sources available for harvesting, namely light, radio frequency (RF) electromagnetic radiation, heat flow, and motion [2,3]. Energy harvesting has received considerable attention regarding the replacement of batteries in wireless power supplies, as well as the recharging of secondary batteries to extend their lifetimes when the energy supply from the environment is intermittent.

Thermoelectric materials convert heat energy into electrical energy via the Seebeck effect [4]. Thermoelectric materials can be used to harvest energy by utilizing temperature differences in the environment. Thermoelectric generators (TEGs) that use heat energy from industrial processes, automobile exhausts, space travel, and human bodies have been developed for energy harvesting [3]. The efficiency of a thermoelectric material is dependent on the dimensionless figure of merit, *ZT* = *S* ^2^ *σT*/*k*, where *S*, *σ*, *k*, and *T* are the Seebeck coefficient, electrical conductivity, thermal conductivity, and absolute temperature, respectively, [5–7]. It is necessary to decrease the thermal conductivity or increase the power factor (*S* ^2^ *σ*) of thermoelectric materials to enhance their efficiency. The most widely used commercial thermoelectric materials are alloys based on the Bi_2_Te_3_ system (*ZT* ~ 1) [8,9]. However, these materials are incompatible with the metal–oxide–semiconductor (CMOS) technology, and not environmentally friendly. Si is the most abundant and environmentally friendly semiconductor material, and can potentially be used to overcome these limitations. Furthermore, owing to the rapidly developing semiconductor industry, Si can be produced at a low cost with a high yield. However, bulk Si is not a good thermoelectric material (*ZT* ~0.01) because of its high thermal conductivity (~150 W m^−1^ K^−1^) [10]. Recently, Si nanowires (SiNWs) with high thermoelectric efficiencies have been reported; their thermal conductivity has been reduced by two orders of magnitude compared to bulk Si without significantly affecting the power factor, and a *ZT* value of 0.6 at room temperature has been achieved [10,11]. The reproducibility of such high *ZT* value has been proved difficult [12]. Nevertheless, SiNWs could potentially be used for thermoelectric applications owing to their low thermal conductivity and preserving electrical conductivity [13].

Using microfabrication techniques, two types of micro-thermoelectric generator (μTEGs) could be fabricated, namely vertical types (out-of-plane) and planar types (in-plane), depending on whether the temperature gradient is applied in the directions perpendicular or parallel to the surface of the substrate [14–22]. In the case of vertical-type SiNW-μTEGs, a large temperature difference can be retained across the vertically aligned SiNWs. However, high fabrication costs are associated with the deep-etching process required for the top-down approach and the well-controlled nanowire growth that occurs during the bottom-up approach. By comparison, in the case of planar μTEGs, the use of Si substrates with high thermal conductivity reduces the temperature difference across the SiNWs, resulting in low efficiency. However, the fabrication costs associated with planar μTEGs are generally lower than those of vertical μTEGs. To obtain high efficiency, the substrate needs to be etched away to form thermally isolated SiNWs, resulting in a high fabrication cost and fragile structure. In general, a high conversion efficiency is required if the heat source is expensive, while power generation density is important when the heat source is inexpensive or free, such as in the case of waste heat. For the energy harvesting of waste heat, the power generation density and fabrication costs are considered more important than the conversion efficiency because the heat energy is essentially obtained free of charge from the environment [23].

**Figure 1.**
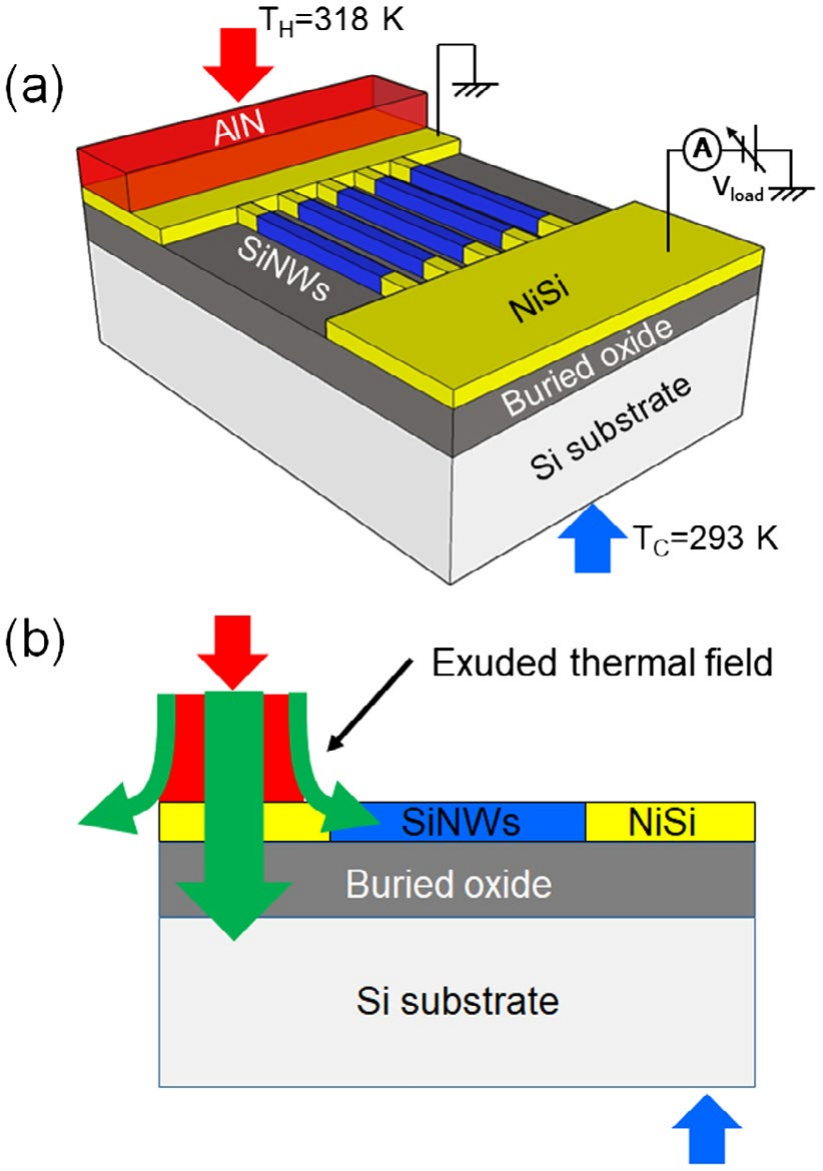
(a) 3D schematic of the proposed planar SiNW-μTEG fabricated on SOI substrates using a CMOS-compatible process. (b) Schematic representation of the exuded thermal field (a steep thermal gradient is generated near the heat source, which decays exponentially along the length of the SiNWs away from the heat source).

In this study, we propose a miniaturized planar SiNW-μTEG architecture, which could be simply fabricated on commercially available silicon-on-insulator (SOI) substrates using the CMOS-compatible top-down process. The Si-μTEG has advantages of low raw material cost of Si and extremely low assembling cost. Furthermore, the processing cost of the proposed device is reduced as compared to conventional μTEGs because there is no need to etch away the substrate to form suspended SiNWs. Figure 1(a) shows a 3D schematic of the proposed SiNW-μTEG. In this SiNW-μTEG architecture, according to our previous simulation results [24,25], the heat flow from the AlN layer penetrates into the Si substrate and an exuded thermal field is formed across the SiNWs (schematically shown in Figure 1(b)). As a result, a steep temperature gradient is generated near the heat source, which decays exponentially along the length of the SiNWs away from the heat source. The exuded thermal field can be used for power generation. Therefore, there is no need to etch away the substrate to form suspended SiNWs that is necessary in the conventional nanowire μTEGs, leading to a low fabrication cost and well-protected SiNWs. We experimentally demonstrated that the power generation density of the SiNW-μTEGs was enhanced by four orders of magnitude when the SiNWs were shortened from 280 to 8 μm. Furthermore, we succeeded in enhancing the thermoelectric power by employing a reactively sputtered AlN film as a thermally conductive layer to reduce parasitic thermal resistance. The thermoelectric power of these SiNW-μTEGs was enhanced by an order of magnitude compared with that achieved when a non-reactively sputtered AlN was used as the thermally conductive layer. We measured the thermal conductivity of the two AlN films. The results indicate that the reduction in the parasitic thermal resistance was caused by an increase in the thermal conductivity of the AlN thermally conductive layer and a decrease in the thermal boundary resistance.

## 2. Materials and methods

### 2.1. Fabrication of SiNW-μTEGs

**Figure 2.**
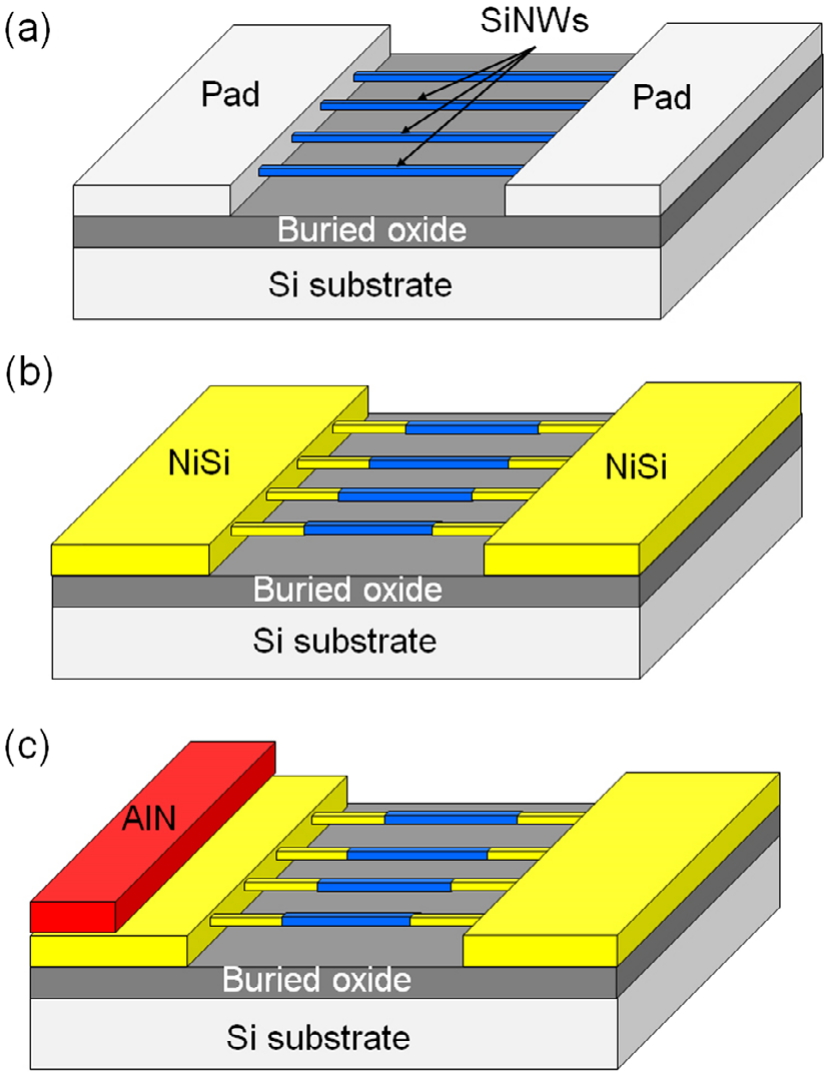
Schematic illustration of the main steps of the process used to fabricate the SiNW-μTEG.

**Figure 3.**
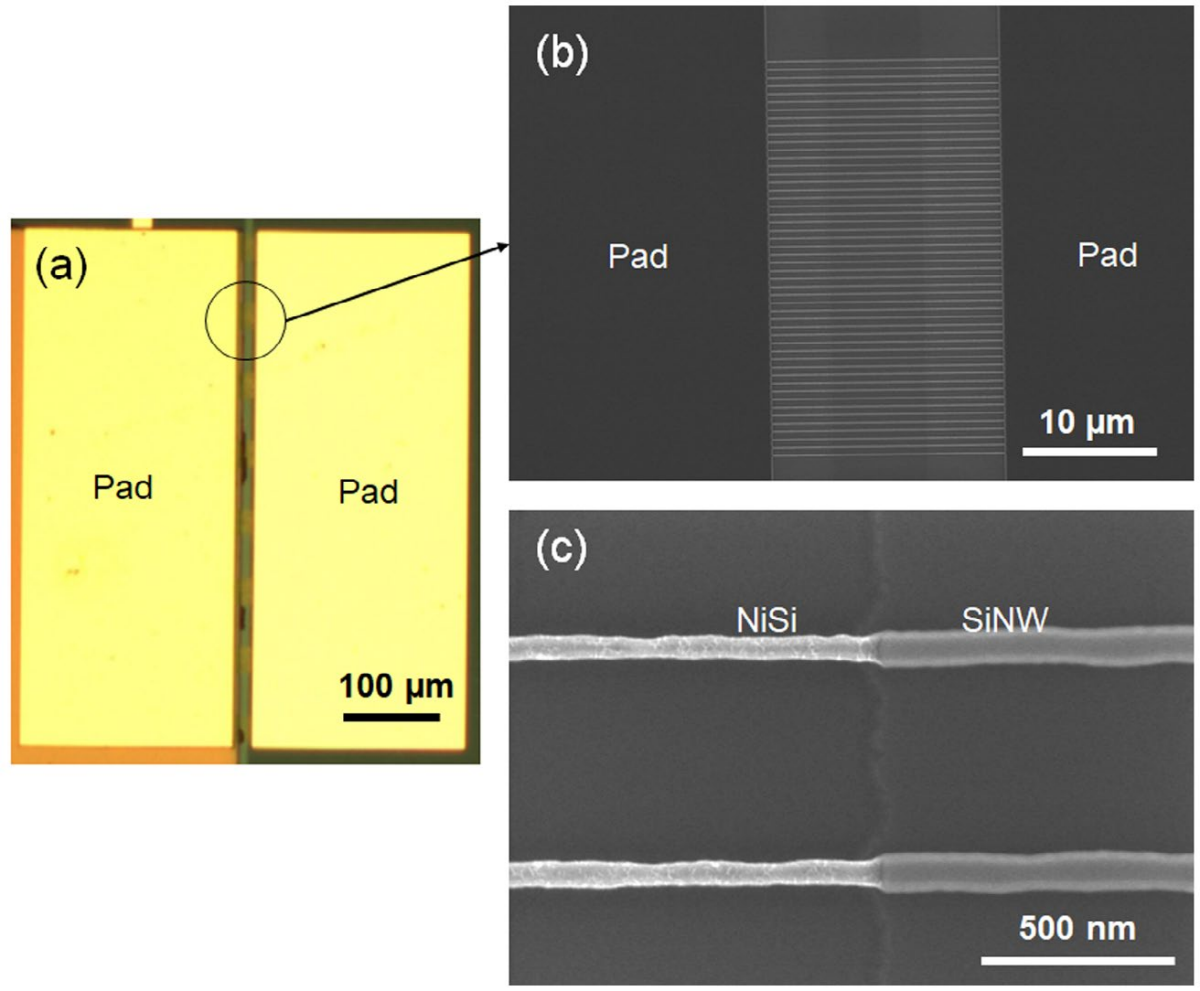
(a) Top-view optical image of the fabricated SiNW-μTEG, which comprises 8 arrays with 400 SiNWs. (b) Top-view SEM image of 1 array with 50 SiNWs. (c) High-resolution SEM image of two individual SiNWs. The SiNWs have a rectangular cross section. The thickness, width, and length of the SiNWs were 35 nm, 100 nm, and 8 μm, respectively.

Figure 2 schematically demonstrates the main steps of the process used to fabricate the SiNW-μTEGs. In this process, SiNWs are patterned onto an SOI substrate via electron-beam lithography and reactive-ion etching (RIE) methods. The thicknesses of the Si substrate, buried oxide (BOX) SiO_2_ layer, and top SOI layer were 750 μm, 145 nm, and 55 nm, respectively. An SAL-601 negative resist was used for the electron-beam lithography. The RIE process was performed with the inductively coupled plasma of a C_3_F_8_/O_2_/Ar mixing gas. Next, thermal oxidation was performed under a dry O_2_ atmosphere at 850 °C for 3 h, to form an SiO_2_ film (thickness ~20 nm) on the surface of the SiNWs. Consequently, the thickness of the SiNWs decreased to approximately 35 nm. Phosphorus (P) ions were then implanted at an acceleration energy of 25 keV, with a dose of 1.0 × 10^15^ ions/cm^−2^. This was followed by activation annealing at 950 °C for 10 min under vacuum conditions. Next, the SiO_2_ layer at the pad regions of the sample was removed via photolithography and a wet-etching process using buffered hydrofluoric acid. Then, the Si pads and both ends of the SiNWs were nickelized by depositing a Ni film (thickness ~20 nm) using magnetron sputtering. This was followed by rapid thermal annealing at 410 °C for 20 min. Both ends of the SiNWs were connected to the NiSi pads. Figure 3 shows the top-view scanning electron microscopy (SEM) images of a part of the fabricated SiNW-μTEG. The fabricated SiNW-μTEG consists of 8 arrays of 400 legs. The SiNWs have a rectangular cross section. The thickness, width, and length of the SiNWs were 35 nm, 100 nm, and 8 μm, respectively. SiNWs with lengths of 46, 90, and 280 μm were also fabricated. Finally, a thermally conductive AlN film was deposited onto one of the NiSi pads via magnetron sputtering to facilitate heat conduction from the heat source. In this study, two deposition methods were utilized to grow the AlN films. One method involved the non-reactive RF sputtering method, where AlN and Ar are used as the sputtering target and sputtering gas, respectively. The other method involved reactive RF sputtering, where Al and an Ar/N_2_ mixture are used as the sputtering target and sputtering gas, respectively. The crystal structures of the AlN films were studied using X-ray diffraction (XRD). The microstructural characteristics of the deposited AlN films were examined using SEM and transmission electron microscopy (TEM). X-ray photoelectron spectroscopy (XPS) was employed to analyze the chemical compositions of the deposited AlN films.

### 2.2. Measurement of thermoelectric properties

**Figure 4.**
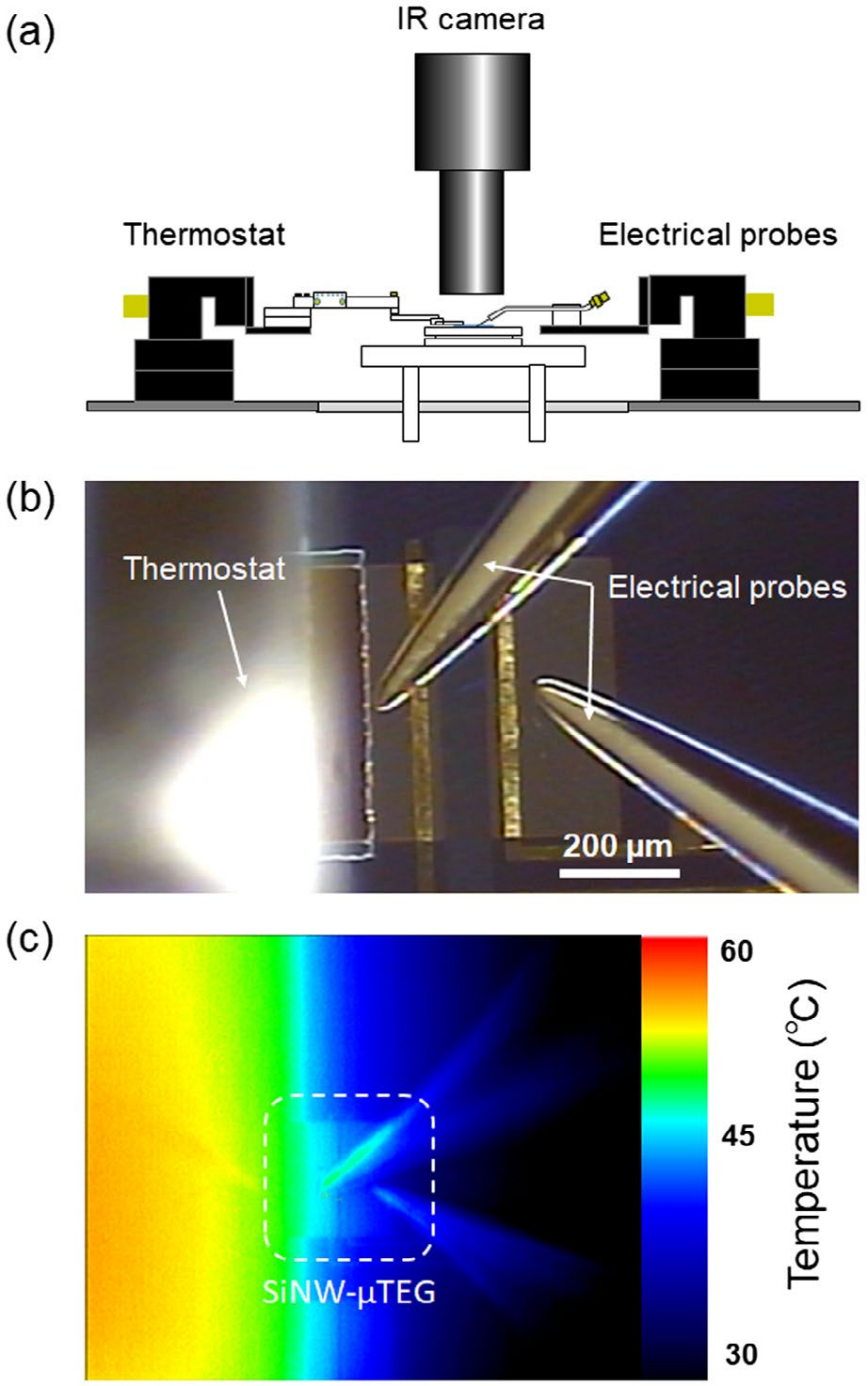
(a) Schematic of the customized measurement system; (b) Optical photograph of the SiNW-μTEG, electrical probes, and thermostat during measurement; (c) Temperature distribution captured by an IR thermography camera when a heat source is applied.

The thermoelectric properties of the SiNW-μTEGs were measured using a customized probe system equipped with an infrared (IR) thermography camera and a thermostat [26]. The thermoelectric properties were measured by applying both a temperature difference and loading voltage to the SiNW-μTEG. The electrical characterization of the SiNW-μTEGs was performed using a semiconductor-device analyzer (Agilent, B1500A, Keysight Technologies, Inc. Santa Rosa, CA, USA). A customized thermostat was used as the heat source, which was placed on the AlN film and maintained at 45 °C. The base of the substrate was maintained at 20 °C, as a cold heat sink. The temperature difference across the SiNWs was measured using the IR thermography camera. The special resolution of the IR thermography camera was 8.4 μm, almost identical to the length of the shortest SiNWs; thus, the measured temperature difference may not be accurate and should be regarded as a rough estimate. Figure 4(a)–(c) show a schematic of the measurement system, an optical photograph of the SiNW-μTEG, the electrical probes, and the thermostat during measurement; and the temperature distribution captured by the IR thermography camera during application of the heat source, respectively.

### 2.3. Measurement of thermal conductivity

The thermal conductivities of the AlN films were measured using the frequency-domain thermoreflectance method (*ω* method) under vacuum conditions (<0.02 Pa) [27]. The AlN films were deposited on Si substrate using the reactive and non-reactive sputtering methods, respectively. The thicknesses of the AlN films were 100, 300, and 500 nm. A 150-nm-thick Au film was then deposited on the AlN film, via sputtering, to function as a laser absorber and temperature sensor. The Au film was heated using a pump laser (405 nm) with an angular frequency, *ω*, of 1, 2, 4, and 8 kHz. The temperature response at the surface of the Au film was measured using a probe laser (635 nm) via the thermoreflectance technique. Measurements were performed at three different locations on each sample; each measurement was performed three times to reduce errors. More details concerning the measurement principles of the *ω* method can be found elsewhere [27–30].

## 3. Results and discussion

### 3.1. Thermoelectric properties of SiNW-μTEGs

**Figure 5.**
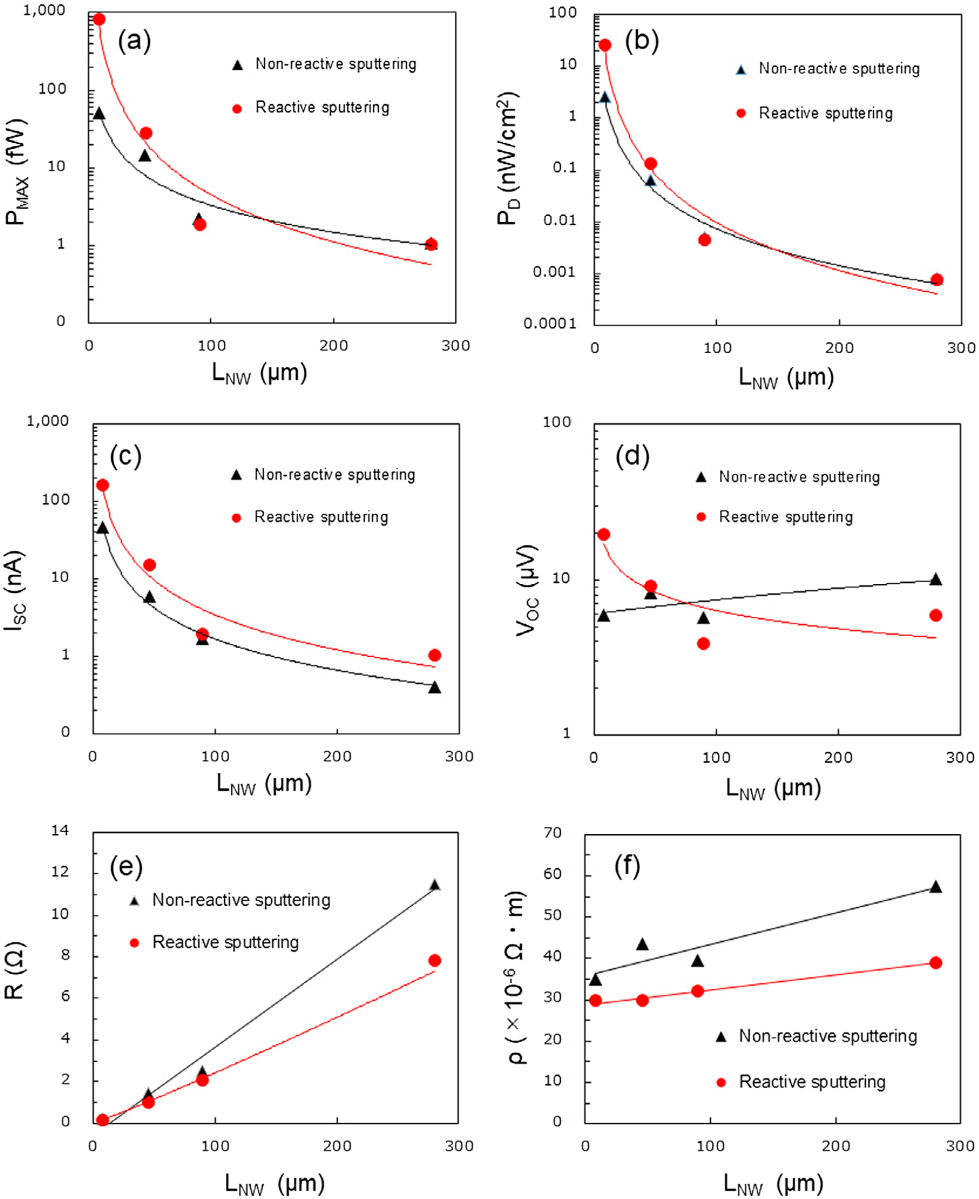
(a) Measured maximum thermoelectric power (*P*_MAX_), (b) maximum power generation density (*P*_*D*_), (c) short-circuit current (*I*_*SC*_), (d) open-circuit voltage (*V*_*OC*_), (e) electrical resistance (*R*), and (f) electrical resistivity (*ρ*) as a function of nanowire length (*L*_*NW*_) when the two different AlN films were used as thermally conductive layers. Solid lines are a guide to the eye.

Figure 5(a)–(d) show the measured maximum thermoelectric power (*P*_MAX_), maximum power generation density (*P*_*D*_), short-circuit current (*I*_*SC*_), and open-circuit voltage (*V*_*OC*_) as a function of nanowire length (*L*_*NW*_), when two different AlN films were used as thermally conductive layers. *P*_*D*_ was calculated by dividing *P*_MAX_ by the total area of the SiNW arrays *A*, which was determined by the following equation. *A* = 2*NdL*_*NW*_, where *N* is the number of the SiNWs, *d* is the pitch of the SiNW arrays. The length of Si pads is assumed to be the same to *L*_*NW*_. For both SiNW-μTEGs with two different AlN thermally conductive layers, the *P*_MAX_ values of the SiNW-μTEGs are very small and only slightly different when *L*_*NW*_ is longer than 90 μm. However, *P*_MAX_ increases significantly as *L*_*NW*_ is shortened to less than 46 μm. When the non-reactively- and reactively sputtered AlN films are used as thermally conductive layers, by shortening the SiNWs from 280 to 8 μm, the *P*_MAX_ values of the SiNW-μTEGs were enhanced by nearly two and three orders of magnitude, respectively. The *P*_*D*_ values were enhanced more than *P*_MAX_ as *L*_*NW*_ was shortened because the total area of the SiNW arrays decreased. Using the reactively sputtered AlN as a thermally conductive layer enhances *P*_*D*_ by an order of magnitude compared with that achieved using the non-reactively sputtered AlN films. *I*_*SC*_ also increases significantly as *L*_*NW*_ is shortened, showing a similar trend to *P*_MAX_. However, *V*_*OC*_ does not show a monotonic increasing trend as *L*_*NW*_ is shortened when the non-reactively sputtered AlN film is used as the thermally conductive layer. When the reactively sputtered AlN film is used as the thermally conductive layer, *V*_*OC*_ increases when *L*_*NW*_ is shortened to less than 90 μm.

We also measured the electrical resistance (*R*) and calculated electrical resistivity (*ρ*) values of the SiNWs with different *L*_*NW*_. Figure 5(e) and (f) show *R* and *ρ* as a function of *L*_*NW*_ when two different AlN films were used as thermally conductive layers. *R* of the SiNWs decreases almost linearly with decreasing *L*_*NW*_, the *ρ* values of the SiNWs with different *L*_*NW*_ are only slightly different. The results indicate that as *L*_*NW*_ is shortened, the significant enhancement in *P*_MAX_ is induced by the significantly decreased *R* and simultaneously unweakened *V*_*OC*_. When the reactively sputtered AlN films are used as thermally conductive layers, the increased *V*_*OC*_ also contributed to the enhancement in *P*_MAX_ in the short SiNWs.

**Figure 6.**
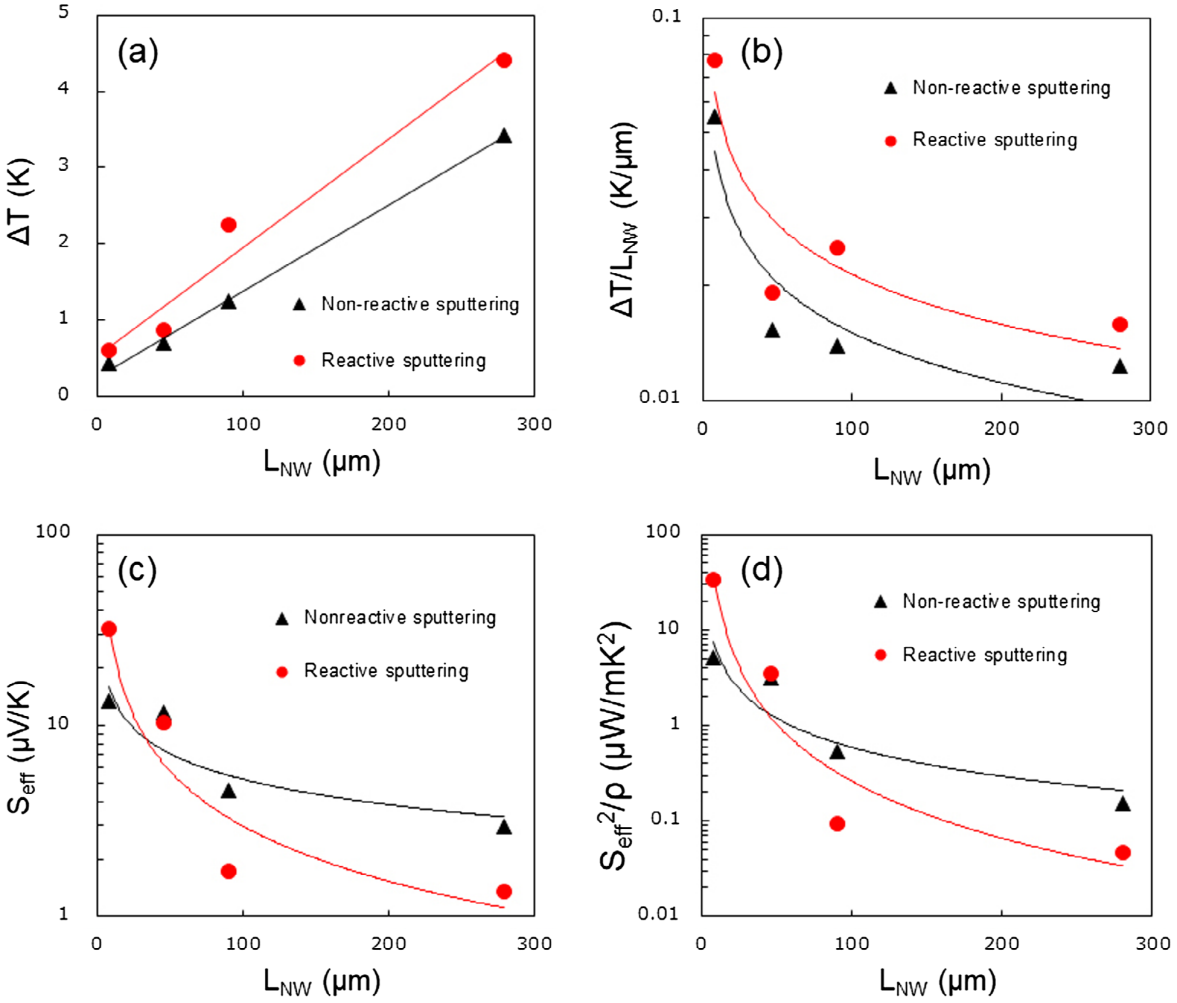
(a) Measured temperature differences (Δ*T*) and (b) thermal gradients (Δ*T/L*_*NW*_) across the SiNWs and (c) the calculated effective Seebeck coefficient (*S*_*eff*_) and (d) power factor ($S_{\mathrm{eff}}^{2}$/*ρ*) of the SiNWs as a function of nanowire length (*L*_*NW*_) when the two different AlN films were used as thermally conductive layers. Solid lines are a guide to the eye.

Figure 6(a) and (b) show the temperature differences (Δ*T*) measured using the IR thermography camera and the thermal gradients (Δ*T/L*_*NW*_) across the SiNWs in the μTEGs. The thermal gradients are calculated by dividing Δ*T* by the corresponding *L*_*NW*_. The temperature difference decreases with a decreasing *L*_*NW*_, but the thermal gradient increases significantly with decreasing *L*_*NW*_ for both SiNW-μTEGs. This is because a steep temperature gradient is generated near the heat source and decays exponentially along the length of the SiNWs away from the heat source. We calculated the effective Seebeck coefficient (*S*_*eff*_) and power factor of SiNWs ($S_{\mathrm{eff}}^{2}$/*ρ*) with different *L*_*NW*_ using *S*_*eff*_ = *V*_*OC*_/Δ*T*. Figure 6(c) shows that *S*_*eff*_ increases with decreasing *L*_*NW*_. Since *ρ* of the SiNWs with different *L*_*NW*_ are only slightly different, $S_{\mathrm{eff}}^{2}$/*ρ* of SiNWs increases more dramatically than *S*_*eff*_ with decreasing *L*_*NW*_ (Figure 6(d)). It is not yet understood why *S*_*eff*_ increases with an increasing Δ*T/L*_*NW*_ as the SiNWs are shortened. The phonon-drag effect [31] and Benedicks effect [32] maybe responsible for the increase in *S*_*eff*_. Elucidating the underlying mechanisms constitutes a future study. We have demonstrated that in the micrometer-scale SiNWs, *P*_MAX_ was significantly enhanced by shortening *L*_*NW*_. Therefore, *P*_MAX_ can be expected to be further enhanced by further shortening *L*_*NW*_ to the nanometer level. Based on our previous simulation results [25], *P*_*D*_ is expected to be enhanced to a mW/cm^2^-class, which can be used to produce electrical power for IoT sensors.

### 3.2. Crystallinity and atomic concentration of AlN films

The temperature differences across the SiNWs of the μTEGs with the reactively sputtered AlN layers are larger than those of the μTEGs with the non-reactively sputtered AlN layers. The total thermal resistance of the μTEGs comprises the thermal resistance of the TEG legs and the parasitic thermal resistance. The two components of the thermal resistance act in series. A relative increase in the parasitic thermal resistance will result in a decrease in the temperature difference across the TEG legs. We thus speculate that the parasitic thermal resistance of the SiNW-μTEGs was reduced when the reactively sputtered AlN was used as a thermally conductive layer, resulting in a larger temperature difference across the SiNWs.

**Figure 7.**
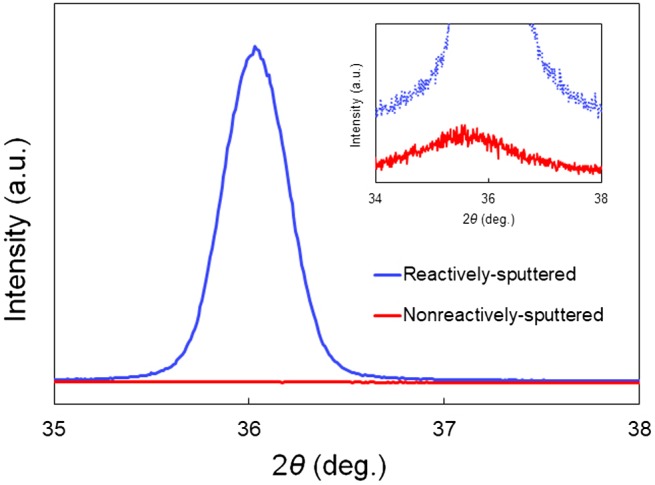
XRD patterns of AlN films deposited by two methods.

We first characterized the crystallinity of the AlN films deposited using the two methods. XRD patterns of the two AlN films are shown in Figure 7. AlN (0002) peaks can be observed in the cases of both AlN films. The reactively sputtered AlN film shows a strong and sharp peak, while the non-reactively sputtered AlN film shows a very weak and broad peak. It is known that the average grain size is inversely proportional to the peak width; this can be determined using the Scherrer equation [33].

$$D=\frac{K\cdot \gamma }{\beta \cdot \cos\theta }$$

where *D* is the average grain size, *K* is a dimensionless shape factor, *γ* is the X-ray wavelength, *β* is the full width at half maximum (FWHM) of the peaks in radians, and *θ* is the Bragg angle. The FWHM values of the AlN (0002) peaks, in the cases of the reactively sputtered and non-reactively sputtered AlN films, were 0.3 and 2.1, respectively. The average grain sizes of the reactively- and non-reactively sputtered AlN films were calculated to be 25 and 3.7 nm, respectively.

**Figure 8.**
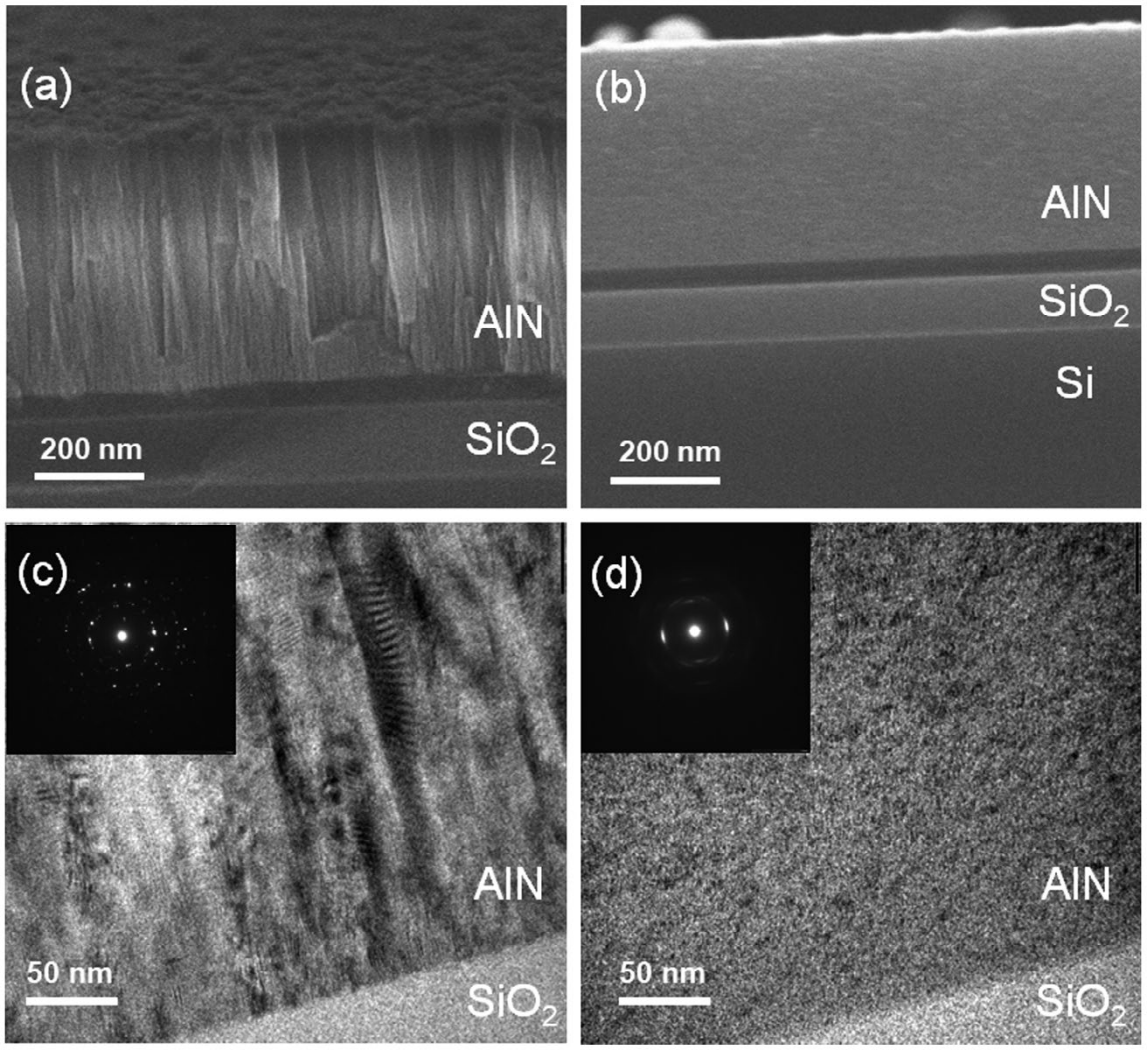
Cross-sectional SEM (a,b) and TEM (c,d) images of the AlN films deposited by reactive sputtering (a,c) and non-reactive sputtering (b,d). The insets show electron diffraction patterns.

We next characterized the microstructures of the AlN films deposited using the two methods by SEM and TEM. Figure 8(a) and (b) show the cross-sectional SEM images of the AlN films deposited using the two methods. The two AlN films have similar thicknesses but exhibit different microstructures. The reactively sputtered AlN film exhibits a polycrystalline structure, and the grain boundaries are aligned perpendicular to the surface of the substrate. The non-reactively sputtered AlN film exhibits an amorphous-like structure; no crystal grains can be observed. The cross-sectional TEM images in Figure 8(c) and (d) show the microstructures of the AlN films more clearly. The reactively sputtered AlN film possesses much larger grains than the non-reactively-sputtered AlN film. In the case of the non-reactively sputtered AlN film, an amorphous-like microstructure is exhibited. The different diffraction patterns also confirm the different crystallinities of the AlN films deposited using the two methods. The XRD, SEM, and TEM results indicate that the crystalline quality of the reactively sputtered AlN film is superior to that of the non-reactively sputtered AlN film.

**Figure 9.**
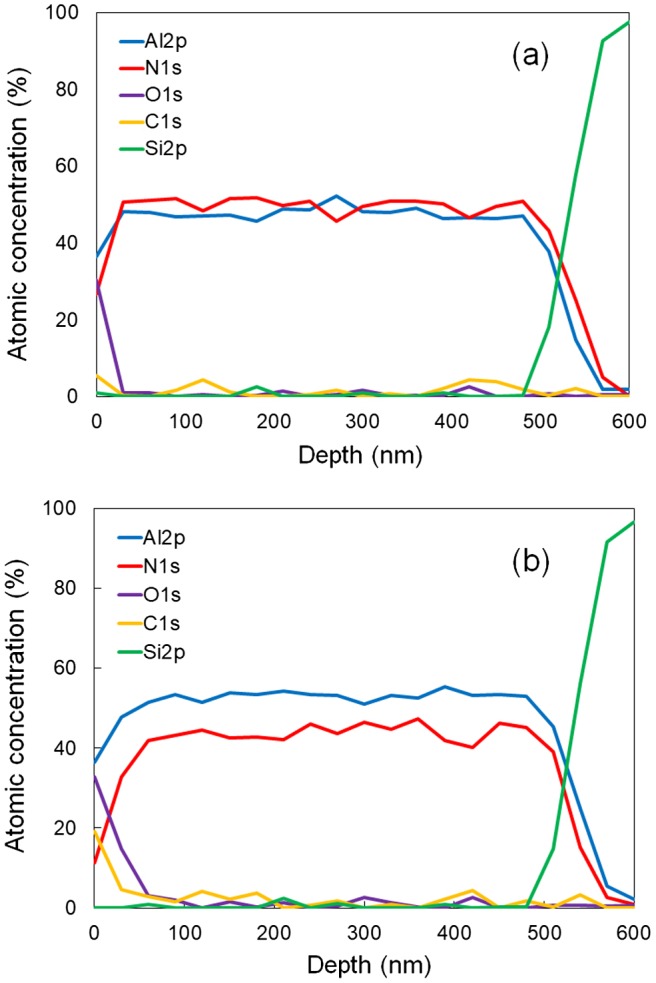
Atomic concentration measured by XPS as a function of depth in AlN films deposited by reactive sputtering (a) and non-reactive sputtering (b).

We then characterized the atomic concentration of the two AlN films by XPS. Figure 9 shows the atomic concentration of various elements, as a function of depth, for the AlN films deposited by the two methods. In both AlN films, oxygen and carbon impurities can be observed near the top surface; this indicates contamination of the surfaces of the AlN films. The atomic composition of the area below the contaminated surface layer primarily consists of Al and N. The reactively sputtered AlN film is stoichiometric, with an Al:N ratio of 1:1. However, in the case of the non-reactively sputtered AlN film, there is a relative increase in the Al concentration, and a slight decrease in the N concentration, resulting in a non-stoichiometric compound with an Al:N ratio of approximately 4.5:5.5. Below the contaminated surface layers of both AlN films, the concentrations of the oxygen and carbon impurities are very low.

### 3.3. Thermal conductivity of AlN films

**Figure 10.**
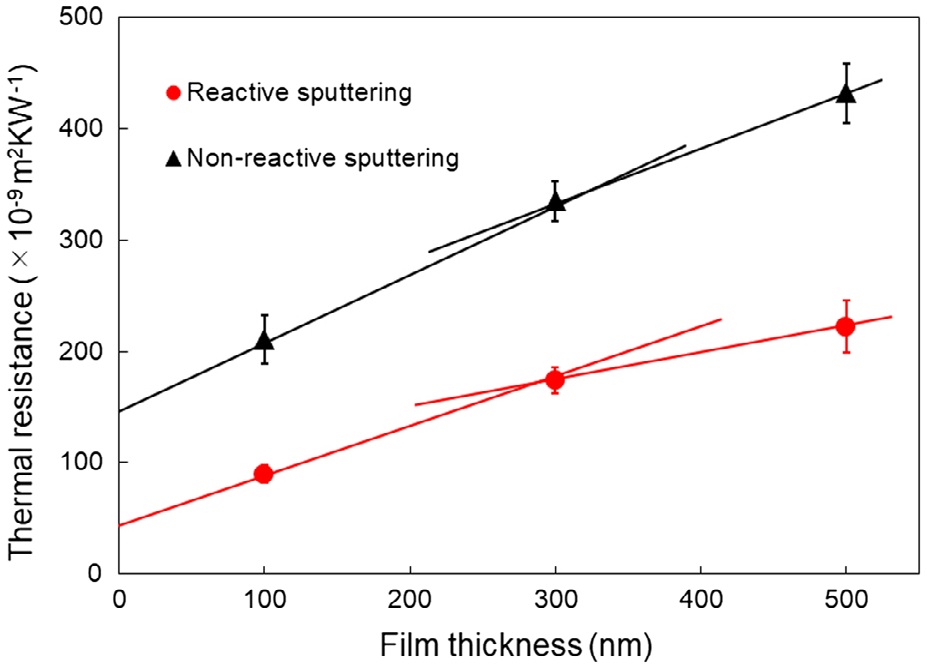
Thermal resistances of Au/AlN/Si as a function of the AlN film thickness. The solid lines are marked by fitting the two data points (100 and 300 nm; 300 and 500 nm).

We next measured the thermal conductivities of the AlN films deposited by the two methods. Figure 10 shows the thermal resistances of Au/AlN/Si as a function of the AlN film thickness. In the case of the reactively sputtered AlN films, the three data points cannot be plotted on a straight line, indicating that the thermal conductivity of the reactively-sputtered AlN films does depend on thickness [34,35]. The thermal conductivity of AlN film increases as the film thickness increases because of the thickness dependence. By fitting two data points (300 and 500 nm), we determined the thermal conductivity of the 500 nm-thick AlN film to be 4.2 W m^−1^ K^−1^, which is significantly lower than that of AlN in bulk form (285 W m^−1^ K^−1^) [36]. This is because thin films have many defects and grain boundaries, which scatter phonons and reduce the thermal conductivity. Furthermore, the scattering of phonons at the film surface limits the phonon mean free paths (MFPs) to the film thickness. The results correlate with the results reported in a previous study, in which the out-of-plane thermal conductivity of reactively sputtered AlN films also exhibited a thickness dependence, and the thermal conductivity of a 500 nm-thick AlN film was determined to be 11 W m^−1^ K^−1^ [33]. Our AlN film has a smaller average grain size than the reported film, which resulted in a lower thermal conductivity. In the case of the non-reactively sputtered AlN films, the three data points are almost on a straight line, indicating that the thickness dependence of the thermal conductivity is weak. By fitting two data points (300 and 500 nm), the thermal conductivity of the non-reactively sputtered AlN film is calculated to be 2.1 W m^−1^ K^−1^. The thermal conductivity results correlate well with the SEM and XRD results, which indicates that the difference in the thermal conductivity is primarily induced by the differences in the crystalline quality of the two AlN films. The slight variation in the atomic composition should have a very weak influence on the thermal conductivity.

The parasitic thermal resistance of the SiNW-μTEGs comprises the thermal resistance of the AlN film and the thermal boundary resistance (TBR) of the AlN/NiSi interface. Based on the measurement principle of the *ω* method, the intercepts of the fitted straight lines represent the total TBR values of the two interfaces (Au/AlN and AlN/Si) of the Au/AlN/Si samples. By fitting two data points (100 and 300 nm), we determined the TBR of the two interfaces. Figure 9 clearly shows that the TBR values of the two interfaces of the non-reactively sputtered samples (1.5 × 10^−7^ m^2^ K W^−1^) are much greater than those of the reactively sputtered samples (4.7 × 10^−8^ m^2^ K W^−1^). In a previous study, the measured TBR values of the Au/AlN and AlN/Si interfaces of an Au/AlN/Si structure were 8 and 7–8 × 10^−8^ m^2^ K W^−1^, respectively, [37]. The reported total TBR values of the two interfaces are almost identical to those of our non-reactively sputtered samples. For comparison purposes, we predicted the TBR using the diffuse mismatch model (DMM) [38]. The speed of sound (longitudinal and transverse), densities, and unit cell volumes of Au, AlN, and Si were taken from a previous study [39]. The DMM-predicted TBR values of the Au/AlN and AlN/Si interfaces were 2.9 and 0.1 × 10^−8^ m^2^ K W^−1^, respectively. The significant difference between the previously measured and DMM-predicted TBR values can be attributed to the interfacial properties of the material, such as the interfacial roughness, interfacial disorder, interfacial attachment, which are not considered in the DMM [40]. We do not know the exact individual TBR value of each interface of our two samples because of limitations associated with the *ω* method. In this study, however, we used an identical Si substrate for both types of samples. In addition, the Au sensing film was deposited under identical conditions for both types of samples. Thus, the difference in the TBR values of the two samples should be attributed to the differences in the crystalline qualities of the two AlN films; this is because the crystalline quality greatly affects the interfacial properties, such as interfacial disorder and interfacial attachment. A previous study reported that the grain size of deposited Au films significantly affects the TBR of Au/sapphire interfaces, and that the TBR of an Au/sapphire interface decreases as the average grain size of an Au film increases [41]. Therefore, in the case of this study, the average grain size of the deposited AlN films should also influence the TBR values of the AlN/Si interfaces. The TBR value of the AlN/Si interface of the reactively sputtered sample, which has a larger average grain size, should be lower than that of the non-reactively sputtered sample.

The results show a relative enhancement in the thermoelectric power of the SiNW-μTEGs with reactively sputtered AlN. This enhancement originates from the improved thermal conductivity of the AlN films and the reduced TBR value of the AlN/NiSi interface. Although the TBR and thermal conductivity of the AlN films were enhanced when using the reactive sputtering method, further improvements can potentially be achieved. To further enhance the thermoelectric power of the SiNW-μTEGs, a thermally conductive film with greater thermal conductivity and a lower TBR should be prepared. For example, the deposition parameters, such as the base pressure, deposition temperature, sputtering power, could be modified to improve the purity and crystalline quality of the thermally conductive films, and the interfacial properties [42].

## 4. Conclusions

We have proposed a miniaturized planar SiNW-μTEG architecture, which can be simply fabricated on SOI substrates using the CMOS-compatible process. Different from the conventional μTEGs, this SiNW-μTEG utilizes an exuded thermal field for power generation. We have experimentally demonstrated that the power generation density of the SiNW-μTEGs was enhanced by four orders of magnitude by shortening the SiNWs from 280 to 8 μm. Furthermore, we succeeded in enhancing the thermoelectric power by employing a reactively sputtered AlN film as a thermally conductive layer to reduce parasitic thermal resistance. We found that the power generation density of the SiNW-μTEGs was enhanced by an order of magnitude compared with that achieved when a non-reactively sputtered AlN film was used as a thermally conductive layer. A power density of 27.9 nW/cm^2^ has been achieved. By measuring the thermal conductivity of the two AlN films, we found that the reduction in the parasitic thermal resistance is owed to the improved thermal conductivity of the AlN film and a reduction in the TBR. All the results indicate that higher power generation densities can be expected by further shortening the SiNWs and reducing parasitic thermal resistance in this μTEG architecture. A mW/cm^2^-class power density is expected based on our previous simulation results [25]. This SiNW-μTEG is a promising energy harvester for wireless power supply in the trillion-sensor network required for the forthcoming IoT society.

## Disclosure statement

No potential conflict of interest was reported by the authors.

## Funding

This work was supported by a CREST [grant number JPMJCR15Q7] from the Japan Science and Technology Agency (JST).
